# A new sagittal parameter to estimate pelvic tilt using the iliac cortical density line and iliac tilt: a retrospective X-ray measurement study

**DOI:** 10.1186/s13018-015-0262-0

**Published:** 2015-07-22

**Authors:** Toshio Doi, Osamu Tono, Kiyoshi Tarukado, Katsumi Harimaya, Yoshihiro Matsumoto, Mitsumasa Hayashida, Seiji Okada, Yukihide Iwamoto

**Affiliations:** Department of Orthopaedic Surgery, Kyushu University Beppu Hospital, 4546 Tsurumi, Beppu, Oita 874-0838 Japan; Department of Orthopaedic Surgery, Graduate School of Medical Sciences, Kyushu University, 3-1-1 Maidashi, Higashi-ku Fukuoka, 812-8582 Japan

**Keywords:** Kyphosis, Iliac bone, Pelvis, Sagittal alignment

## Abstract

**Background:**

When spinal kyphosis increases, the compensatory mechanism activates and the pelvic position changes. Increasing the pelvic tilt, which is the orientation of the pelvis with respect to the femoral head, is known to associate with the clinical symptoms in kyphosis in the aging population. It is often difficult to detect the femoral head on radiographs, limiting the ability to determine the pelvic tilt. Therefore, there is a need to establish another parameter independent of the femoral head which closely correlates with the pelvic tilt.

**Methods:**

Eighty-two adult patients with full-length lateral standing spine radiographs were recruited (mean age: 73.0 years). A new parameter, the iliac cortical density line (a component of the arcuate line of the ilium) and the iliac tilt (defined as the angle between the iliac cortical density line and the vertical), was analyzed to determine the correlation with the pelvic tilt.

**Results:**

Both the pelvic tilt (PT) and iliac tilt (IT) could be identified in 67 patients, and a significant correlation was observed between the PT and IT (*r* = 0.86, *P* < 0.0001). The PT could be estimated using the following formula: PT = IT − 12.9 (in females), PT = IT − 16.7 (in males).

**Conclusions:**

The iliac tilt, which can be easily and directly measured using the iliac cortical density line, is a new parameter that can reliably estimate the pelvic tilt even when the femoral head is not detectable on the radiograph.

## Background

**Fig. 1:**
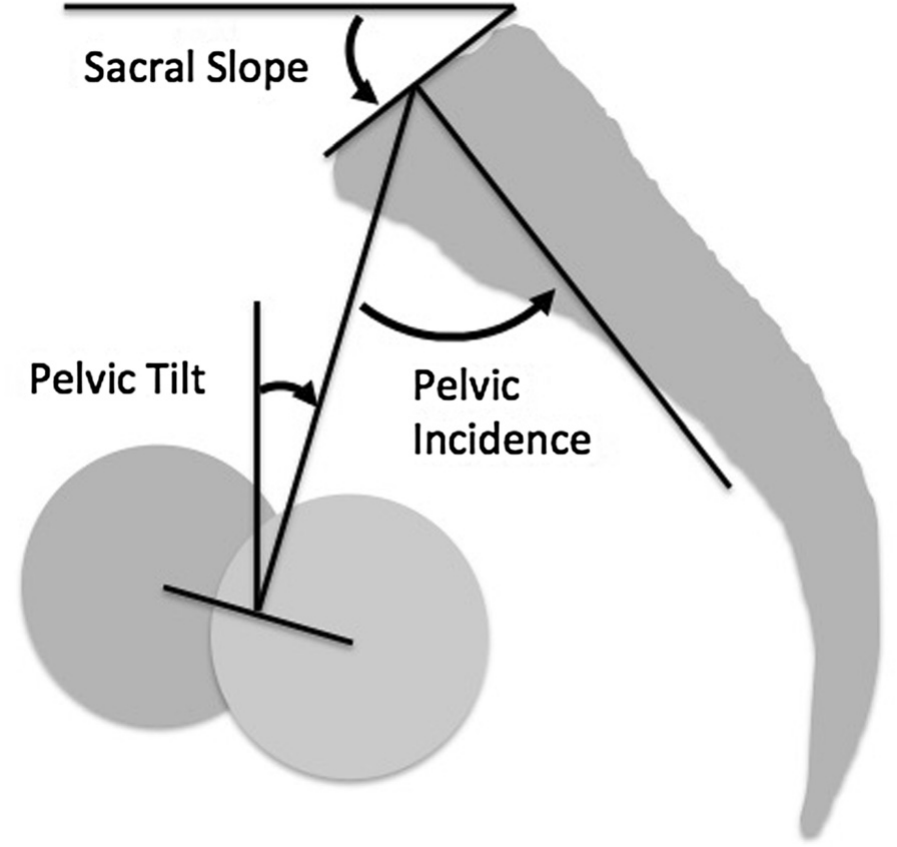
Pelvic parameters. The figure was modified from ref. [2]

The sagittal spinal alignment measurement is becoming important in daily practice. To evaluate the spinal alignment, several spinal and pelvic parameters have been proposed, such as the pelvic incidence (PI), pelvic tilt (PT), lumbar lordosis (LL), and sacral slope (SS) (Fig. 1) [1–5]. The PI is an inherent anatomical parameter and has a strong influence on the LL. The PT and SS are positional parameters, related to the position of the spinal sagittal curvature and the sagittal inclination of the pelvis.

During the progression of spinal kyphosis, the compensatory mechanism activates and the PT increases. Recently, according to the worsening of the sagittal alignment in kyphosis patients, the PT has been demonstrated to affect the clinical outcomes [5].

**Fig. 2:**
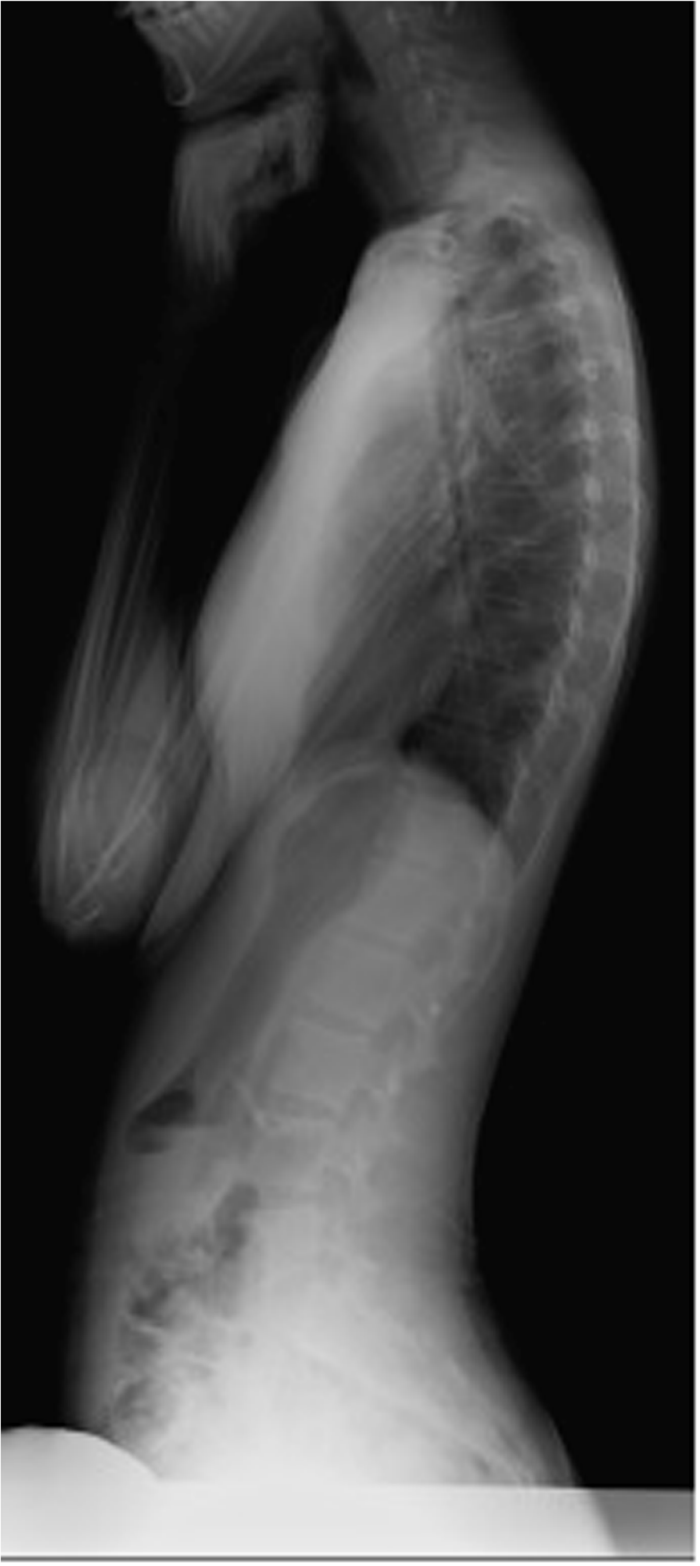
Representative lateral X-rays demonstrate that the evaluation of the femoral head is not possible in the lateral view

The PT can be calculated by the radiographic measurement. However, in the cases where the femoral head is out of view on the lateral film or cannot be identified, the PT cannot be defined (Fig. 2). Recently, it was reported that the sacro-femoral-pubic angle, as a coronal parameter, can reliably estimate pelvic tilt [6].

**Fig. 3:**
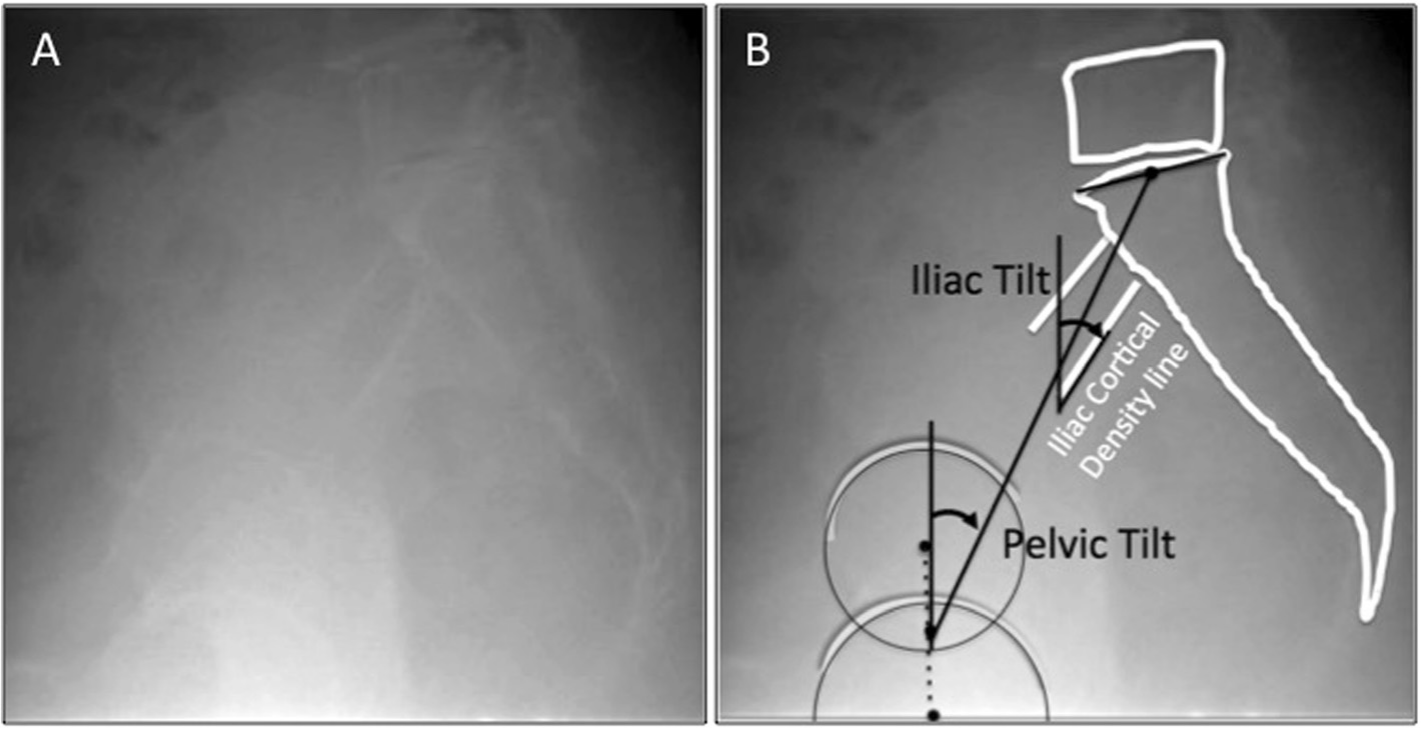
Iliac cortical density line and the iliac tilt. In the lateral X-ray, the iliac cortical density line can be seen as a *straight line* several centimeters in length around the anterior border of S1, which is considered to reflect the thickness of the cortical bone of the ilium along with the arcuate line of the ilium (**a**). The iliac tilt (IT) was defined as the *angle* between the lower (the farther side to the film) iliac cortical density line and the vertical (**b**)

**Fig. 4:**
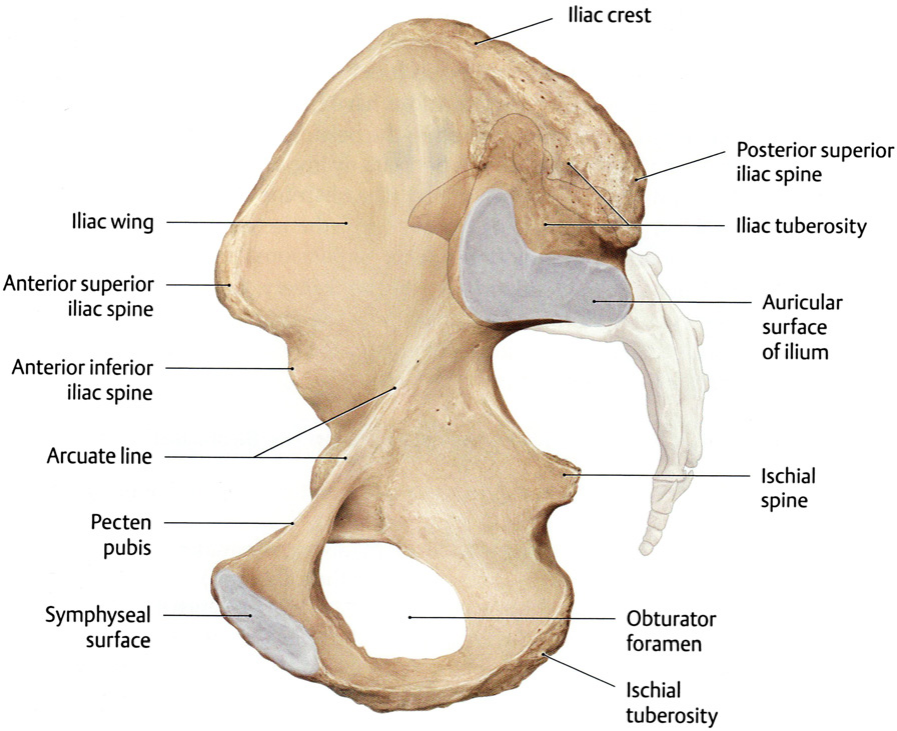
Arcuate line of the ilium. The pelvis is divided by an oblique plane passing through the prominence of the sacrum, the arcuate and pectineal lines, and the upper margin of the symphysis pubis, into the greater and the lesser pelvis (the figure is taken from Thieme Anatomy)

On the lateral X-ray, the iliac cortical density line can be seen as a straight line several centimeters in length around the anterior border of S1 (Fig. 3), which is considered to reflect the thickness of the cortical bone of the ilium along with the arcuate line of the ilium (Fig. 4). We thought that the iliac cortical density line might be another pelvic anatomical parameter to evaluate the spinal alignment. The purpose of this study is to describe a new sagittal pelvic parameter (referred to as the iliac tilt (IT)) and to evaluate its correlation with the PT.

## Methods

### Study design

This study was a retrospective review of 82 adult patients (52 females and 30 males) who underwent full-length lateral spine radiographs for clinical purposes at our institute. The exclusion criteria were the presence of a pelvic osseous abnormality, severe osteoarthritis of the hip, and previous total hip replacement. The patient background information and underlying diseases are shown in Table 1.

**Table 1:** The underlying diseases in the patients

Type of disease	Number
Lumbar spinal canal stenosis	33
Cervical spondylosis	14
Vertebral column fracture	12
Spinal kyphosis	7
Low back pain	4
Ossification of the ligamentum flavum	4
Spondylolisthesis	3
Lumbar disc herniation	3
Dropped head syndrome	1
Knee osteoarthritis	1
Total	82

### Radiographic measurements

Spinal and pelvic parameters were measured on long-cassette standing upright lateral radiographs. All radiographs were taken in the fists-on-clavicle position. The PT was calculated using a digital analysis software program (Fuji Synapse System, Fujifilm holdings, Tokyo, Japan). Anatomically, the right and left cortical density lines exist, however, because the intensity of the line is dependent on the X-ray incident angle, the iliac cortical density line of the farther side to the film is thicker than the closer side to the film, and only the farther side to the film can be found in many cases. If both iliac cortical density lines can be observed, the closer side to the film is detected as the upper line and the farther side to the film is detected as the lower line. The IT was defined as the angle between the lower iliac cortical density line and the vertical (Fig. 3).

### Interobserver and intraobserver reliability

Three orthopedic surgeons (observer 1, observer 2, and observer 3) were familiarized with the computer program and also taught how to measure the IT on the computer monitor. The measurements were carried out twice on different occasions with 12 radiographs. Four weeks passed between the measurements. The interobserver and intraobserver agreement was assessed by the interclass correlation coefficient.

### Statistical analysis

The relationship between the PT and IT was evaluated using Pearson’s coefficient of correlation and the linear regression model. A subgroup analysis by sex was conducted to evaluate the influence of gender on these parameters. The statistical analysis was conducted using the Prism software program (GraphPad Software, CA, USA) with a level of significance set at 0.05.

## Results

### Patient demographics

Eighty-two patients were recruited in this study with a mean age of 73.0 years (range: 31–88 years). Fifty-two patients were females and 30 were males. The mean age was 75.2 for the males and 69.6 years for the females.

### Interobserver and intraobserver reliability

The interclass correlation coefficient for the interobserver reliability of the IT measurements using lateral radiographs by the three observers was 0.87 (Table 2). The interclass correlation coefficient for the intraobserver reliability of the IT measurements using lateral radiographs was 0.89 for observer 1, 0.87 for observer 2, and 0.87 for observer 3 (Table 3).

**Table 2:** The interobserver reliability analysis for the measurement of the IT. Each observer measured the IT twice, on different occasions (*N* = 36), and the interclass correlation coefficient was calculated

	Interclass correlation coefficient	95 % confidence interval
IT angle measurement (*N* = 36)	0.87	0.77–0.93

**Table 3:** The intraobserver reliability analysis for the measurement of the IT. Each observer measured the IT (*N* = 12) and the interclass correlation coefficient was calculated. The numerical value shows the gamma of the interclass correlation coefficient and the range of the 95 % confidence interval

	Interclass correlation coefficient
Observer 1 (*N* = 12)	0.89 (0.79–0.94)
Observer 2 (*N* = 12)	0.87 (0.76–0.93)
Observer 3 (*N* = 12)	0.87 (0.77–0.93)

### PT and IT

The PT could not be identified in 6 patients due to the loss of identification of the femoral head in 4 patients and lumbosacral transitional vertebra in 2 patients. The mean PT was 21.9° ± 10.2°(mean ± SD) (Table 4).

**Table 4:** Summary of the mean PT and PI angles among the groups. No statistically significant differences were observed between the males and females

	Number	PT		IT	
		Mean	SD	Mean	SD
Females	41	23.7	10.9	36.6	9.5
Males	26	19.6	9.1	36.3	7.9
Total	67	21.9	10.2	36.3	8.7

For measuring the IT, both cortical density lines were identified in 16 patients, and the difference angle between the upper and lower iliac cortical density lines was 1.6° ± 3.7° (mean ± SD). In 57 patients, only one cortical density line could be identified. In 9 patients, the iliac cortical density line could not be identified. The mean IT (calculated using the lower IT) was 36.3° ± 8.7° (mean ± SD) (Table 4). There were no statistical differences in the PT and IT between males and females.

### Correlation analysis and linear models

**Fig. 5:**
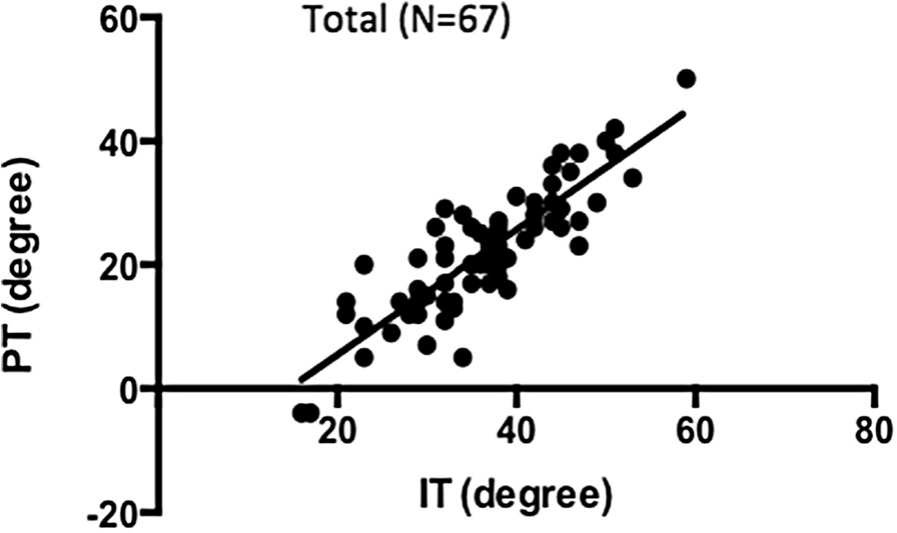
Correlation between IT and PT. There was a statistically significant correlation between the IT and PT (*ρ* = 0.86, *P* < 0.0001, Pearson’s correlation coefficient)

Both the PT and IT could be identified in 67 patients (41 females and 26 males), and a correlation analysis was performed in those patients (Fig. 5, Table 5). The statistical analysis between the IT and PT demonstrated Pearson’s correlation coefficient of *r* = 0.86 (*P* < 0.0001). By a linear regression analysis, R square was 0.744, and the PT could be predicted using the following equation: PT = 1.01 × IT − 14.7. Because the difference between the IT and PT was 14.4° ± 5.2° (mean ± SD), the prediction formula could be simplified as follows: PT = IT − 14.4.

**Table 5:** Summary of the statistical results. A correlation between the IT and PT was observed. Furthermore, the difference between the IT and PT was significantly larger in the males than the females

	Pearson’s coefficient r	Predicted PT with the simplified formula
Females	0.85 (*P* < 0.0001)	PT = IT − 12.9
Males	0.93 (*P* < 0.0001)	PT = IT − 16.7
Total	0.86 (*P* < 0.0001)	PT = IT − 14.4

The difference between the IT and PT in females was 12.9° ± 5.7° (mean ± SD), and in males it was 16.7° ± 3.3° (mean ± SD). The difference between the IT and PT was significantly larger in males than in females (*P* = 0.03). In females, according to the linear regression analysis, R square was 0.725, and the PT could be predicted using the following equation: PT = 0.977 × IT − 12.1. Because the difference between the IT and PT was 12.9° ± 5.7° (mean ± SD), the prediction formula could be simplified as follows: PT = IT − 12.9. In males, according to the linear regression analysis, R square was 0.871, and the PT could be predicted using the following equation: PT = 1.072 × IT − 19.3. Because the difference between the IT and PT was 16.7° ± 3.3° (mean ± SD), the prediction formula could be simplified as follows: PT = IT − 16.7.

## Discussion

Kyphosis deformity is known to have a significant correlation with the worsening of clinical symptoms [4, 5]. The pelvic positional change is prominent during the compensation stage of kyphosis, and the PT is a reliable parameter to detect the pelvic positional change [5]. The PT is the inclination of the line to the vertical between the sacrum to the middle of the center of the femoral head [2]. Recently, the PT has been applied as a surgery reference [7–9]. However, there are some cases where the femoral head cannot be identified on the lateral X-ray. Thus, there is an interest to have alternative pelvic parameters that correlate closely with PT in order to provide a solution for evaluation of pelvic position when measurement of PT is impossible on the X-rays.

The arcuate line of the ilium is an anatomical structure between the sacrum and femoral head. The pelvis is divided by an oblique plane passing through the prominence of the sacrum, the arcuate and pectineal lines, and the upper margin of the symphysis pubis, into the greater and the lesser pelvis [10]. On the lateral X-ray, the iliac cortical density line can be seen as a line several centimeters in length from the anterior border of S1, which is considered to reflect the thickness of the cortical bone of the ilium along with the arcuate line. Theoretically, two cortical density lines exist, the right and left. On the lateral X-ray, the cortical density line closer to the film cannot be detected in many cases. Typically, the cortical density line farther from the film, which is recognized on the lateral X-ray as the lower line compared to the other side, is readily observed because of the X-ray incident angle. The two cortical density lines do not show the same angle, however, the differences between the two are not large (i.e., −2° to 8°, average 1.6°, in 16 patients). We therefore believe that it is practical to use the lower cortical density line, which is farther from the film.

There may be some disadvantages of measuring the iliac cortical density line. The cortical density line may slightly curve, especially in the anterior border of S1. In those cases, the error of measurement may increase. Among 82 patients, neither sides of the iliac cortical line could be detected in 9 patients. Several conditions for the inability to detect the line may be obese patients, patients with abundant bowel gas, and patients with an abnormal anatomical pelvic shape. In addition, the cortical density line is affected by the conditions of taking the X-ray, which may be particularly affected by the incident angle.

Finally, we also demonstrated that the IT, which is the angle of the iliac cortical density line to the vertical, was significantly correlated with the PT. The IT can be measured in the patients whose PT cannot be identified, such as those with lumbosacral transitional vertebra, severe deformity of the femoral joint, or an unidentifiable femoral head on the X-ray.

## Conclusion

The IT, which can be easily and directly measured using the iliac cortical density line, is a new parameter that can reliably estimate the PT, even when the femoral head is not detectable on the radiograph.

### Endnotes

Portions of this work were previously presented in abstract form at the Combined Academic Conference of the 18th Biennial Congress of Asia Pacific Orthopaedic Association and the 36th Annual Meeting of the Royal College of Orthopaedic Surgeons of Thailand, Pattaya, Thailand, 2014.

## Abbreviations

PI: pelvic incidence
PT: pelvic tilt
LL: lumbar lordosis
SS: sacral slope
IT: iliac tilt
